# Posterior Lumbar Interbody Fusion Using a Unilateral Single Cage and a Local Morselized Bone Graft in the Degenerative Lumbar Spine

**DOI:** 10.4055/cios.2009.1.4.214

**Published:** 2009-11-25

**Authors:** Dong-Hee Kim, Soon-Taek Jeong, Sang-Soo Lee

**Affiliations:** Department of Orthopaedic Surgery, Gyeongsang National University School of Medicine, Jinju, Korea.

**Keywords:** Spinal fusion, Posterior lumbar interbody fusion, Unilateral single cage, Local morselized graft

## Abstract

**Background:**

We retrospectively evaluated the clinical and radiological outcomes of posterior lumbar interbody fusion (PLIF) with using a unilateral single cage and a local morselized bone graft.

**Methods:**

Fifty three patients who underwent PLIF with a unilateral single cage filled with local morselized bone graft were enrolled in this study. The average follow-up duration was 31.1 months. The clinical outcomes were evaluated with using the visual analogue scale (VAS) at the pre-operative period, at 1 year post-operation and at the last follow-up, the Oswestry Disability Index, the Prolo scale and the Kim & Kim criteria at the last follow-up; the radiological outcomes were evaluated according to the change of bone bridging, the radiolucency, the instablity and the disc height.

**Results:**

For the clinical evaluation, the VAS pain index, the Oswestry Disability Index, the Prolo scale and the Kim & Kim criteria showed excellent outcomes. For the the radiological evaluation, 52 cases showed complete bone union at the last follow-up. Regarding the complications, only 1 patient had cage breakage during follow-up.

**Conclusions:**

PLIF using a unilateral single cage filled with a local morselized bone graft has the advantages of a shorter operation time, less blood loss and a shorter hospital stay, as compared with the PLIF using bilateral cages, for treating degenerative lumbar spine disease. This technique also provides excellent outcomes according to the clinical and radiological evaluation.

For patients who suffer with degenerative lumbar spine disease and who present with chronic back pain and neurological symptoms, decompression and spinal fusion surgeries such as anterior lumbar interbody fusion, posterior lumbar interbody fusion (PLIF) and posterolateral fusion are solutions for treating the pain and spinal instability. Anterior lumbar interbody fusion and PLIF are regarded as the most satisfying techniques.^1^-4)

PLIF, which was popularized by Cloward in the early 1950s, has advantages for restoration of the disc height, disc stabilization, nerve root decompression and reinforcement of the anterior spinal column, which is the weight-bearing axis.^5^,6) In PLIF, two cages are usually inserted via a bilateral approach with extensive laminectomy or posterior facetectomy, and segmental pedicle screw fixation is additionally performed to prevent iatrogenic instability of the posterior joint.^7^-9) Unfortunately, the extensive exposure required for circumferential fusion can cause unnecessary trauma to the posterior lumbar or the posterolateral musculoligamentous complex, and this can result in unsatisfactory clinical outcomes.^9^-11)

The bone grafts used in PLIF should have an inherent osteogenic capacity and good mechanical strength. Autogenous iliac bone is the most proper graft in terms of osteogenic capacity, but it is associated with donor site pain and additional surgical invasion, while allogeneic bone grafting carries a risk of infection. Against this backdrop, local bone grafting was introduced as an alternative.^12^-15) However, it is not preferred by many surgeons because of its questionable osteogenic capacity.

In this study, we performed unilateral PLIFs using a single titanium cage, which is known for its high menchanical strength and biocompatibility. Each cage was filled with a local morselized bone graft that was composed of the lamina, the articular process and the spinous process obtained during posterior decompression. We retrospectively analyzed the clinical and radiographic outcomes of this technique.

## METHODS

### Materials

Between January 2003 and September 2006, unilateral PLIFs using a single cage filled with a local morselized bone graft were performed at our institution for the patients who were diagnosed with spinal stenosis, herniation of an intervertebral disc combined with lumbar instability, and spondylolisthesis. The local chip bone graft, which was obtained during posterior decompression, was packed in the anterior area before cage insertion and after performing discectomy. Fifty three of these patients who were followed up for more than 1 year were included in this study. The mean age at the time of surgery was 59.1 years (range, 39 to 77 years). There were 18 males and 35 females. The mean follow-up period was 31.1 months (range, 12 to 54 months). The indication for surgery was spinal stenosis in 36 cases, spondylolisthesis in 12 and herniation of an intervertebral disc combined with lumbar instability in 5. Single-level fusion was perfomed in 39 patients, two-level fusion was done in 9 and three-level fusion was done in 5.

### Surgical Technique

**Fig. 1:**
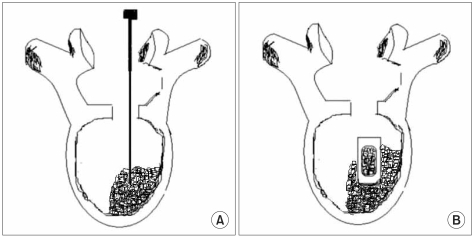
Diagrams depicting the steps of the posterior lumbar interbody fusion via a unilateral approach. (A) After the retraction of the thecal sac and the traversing nerve root to the midline, the disc material and endplates were removed as much as possible in the ipsilateral side. Before the cage insertion, the local morselized bone from the decompressed lamina, spinous process and facets was grafted as much as possible into the ipsilateral and anterior side of the intervertebral space. (B) The single cage filled with local morselized bone graft was introduced to the intervertebral space. Last, adequate impaction was performed.

The patients were placed in the prone position under general anesthesia. With the muscles adjacent to the spine retraced laterally to minimize damage, the area lateral to the lamina and the posterior joint was exposed via a posteromedial approach while the transverse process was not exposed. Nerve root decompression was achieved by performing laminectomy, complete excision of the inferior articular process and discectomy, depending on the cause of disease. The insertion of a single cage was planned on the side with the more severe symptoms or the more severe stenotic foramen seen on MRI, and the dura mater and the nerve root were medially retracted. Extensive removal of the intervertebral disc and the adjacent end plates was performed on the ipsilateral side with using a pituitary rongeur and a curved curette until subchondral bone was exposed. The size of a cage was determined based on the disc height. The involved titanium cages (Titanum O.I.C.®, Stryker, NJ, USA) were of various sizes (width: 11 mm, angulation: 0°, 4°, 8°, height: 9-13 mm, length: 20, 25 mm) and they were rectangular in shape and radiopaque. The lamina, spinous process and posterior articular process obtained during decompression were morselized in a bone mill and packed into the cage. Before the insertion of the cage, the local morselized bone was grafted as much as possible into the anterior side of the intervertebral space (Fig. 1). Pedicle screw fixation was carried out after inserting the cage to secure the stability and to improve the bony union immediately after surgery. Standard wound closure was performed following hemostasis. From the 3rd postoperative day, a lumbo-sacral orthosis was used for 4-5 weeks postoperatively when the patient was walking.

### Assessments

The clinical evaluation was based on the visual analogue scale (VAS) for the preoperative back pain and radiating pain at the 1st , 2nd, 4th, 6th, 12th, and 24th postoperative month, and at the last follow-up. The Oswestry Disability Index was assessed preoperatively and at the last follow-up, the Prolo scale was obtained at the last follow-up and the Kim & Kim criteria16) were also used (Tables 1 and 2).

The lateral plain radiographs taken preoperatively, immediately postoperatively and at the last follow-up were compared for the radiological assessment. Although the radiopaque titanium cage made it difficult to assess whether boney union was achieved, the local morselized bone graft impacted anterior to the cage allowed for directly evaluating the boney union. In other words, we carefully looked for bone bridging and radiolucency around the cage and the metal screws, and any evidence of instability on the flexion-extension lateral radiographs for assessing the boney union. The changes in the intervertebral disc height were evaluated using the restored disc height, based on the measurements performed preoperatively, immediate-postoperatively and at the last follow-up. The tube-to-patient distance was 40 inches and any magnification error was avoided by adjusting the radiation dose of the anteroposterior and lateral radiography to 5.50 dGycm^2^ and 10.00 dGycm^2^, respectively. The boney union status was classified into solid union, delayed union and non-union. Solid union was considered to be obtained when the endplates observed immediately postoperatively on the radiographs became invisible during the follow-up examinations and there was bony trabecular continuity and bone bridging from the graft to the adjacent vertebral bodies in the intervertebral space, the bone graft that appeared as granules on the lateral radiographs became a radiopaque mass after union and any instability on the flexion-extension radiographs and radiolucencey around the cage and screws were not observed. Non-union was defined as disruption of the trabecular continuity, the appearance of instability on the flexion-extension radiographs and ≥ 1 mm radiolucency around the screws and cage. Delayed union was diagnosed when all of the definitions of solid union were met despite that disruption of the trabecular continuity and evidence of non-union were not observable.17) Instability was considered present when ≥ 3° of posterior angular formation was observed on the lateral radiographs and ≥ 2 mm of displacement of the vertebral body and cage movement occurred.

The duration of surgery and the hemorrhage volume were also recorded.

SPSS ver. 12.0 (SPSS Inc., Chicago, IL, USA) was used for the statistical anaylsis. The changes in the intervertebral disc height were evaluated using a paired-sample t-test with a 95% confidence interval.

## RESULTS

The clinical outcomes were as follows. The VAS score measured preoperatively, at the 1st postoperative year and at the last follow-up examination improved significantly to 6.5, 2.8 and 1.8, respectively, for the back pain and to 6.1, 2.7 and 1.8, respectively, for the radiating pain (Table 3). The Oswestry Pain Index remarkably improved from 70.0 preoperatively to 37.9 at the last follow-up and the Economic Prolo Scale was 3 in 3 cases, 4 in 38 cases and 5 in 12 cases while the Functional Prolo Scale was 3 in 5 cases, 4 in 36 cases and 5 in 12 cases; according to the Kim & Kim criteria, 12 (23%) of the 53 cases had a score of excellent, 39 (73%) good, and 2 (4%) fair cases.

**Fig. 2:**
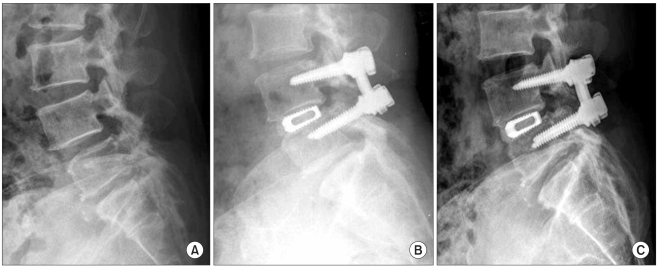
A 51-year old female with spinal stenosis at L4-5. (A) The preoperative lateral view shows a decreased disc height at L4-5. (B) The lateral view after surgery shows restoration of the disc height. (C) At 3 year after surgery, the lateral radiograph shows solid fusion and maintenance of reduction.

**Fig. 3:**
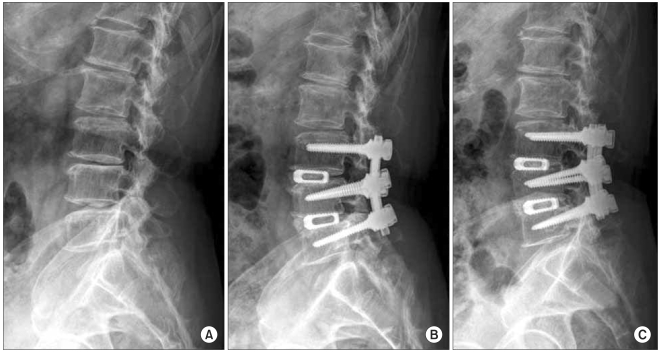
A 71 year-old male who presented with lower back pain and motor weakness. (A) The lateral radiograph shows disc space narrowing on L3-4-5 and degenerative kyphosis. (B) The posterior lumbar interbody fusion with a single cage and a transpedicular instrument was performed and the follow-up radiograph shows maintenance of the disc height and restoration of lordosis. (C) At 3 years after surgery, the lateral radiograph shows solid fusion and maintainance of the reduction.

**Fig. 4:**
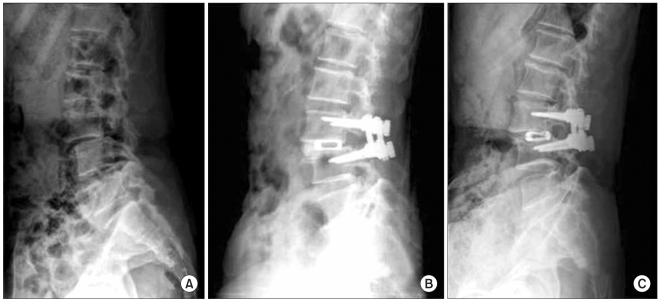
A 53 year-old female who presented with a 1-year history of lower back pain and radiating right leg pain. (A) The lateral radiograph shows spondylolisthesis on L3-4. (B) The lateral view after surgery shows restoration of the disc height. (C) The last follow-up radiograph shows breakage of the cage, but the disc space was maintained and there is probable bone bridging without radiolucency and with stability on the flexion and extension views.

**Fig. 5:**
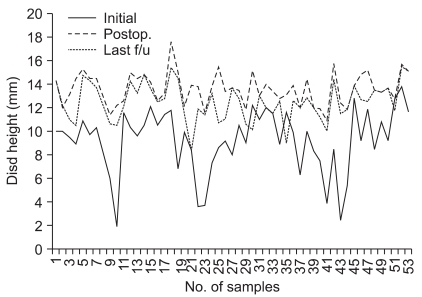
Changes of the disc height according to the preoperative, immediate postoperative and last follow-up.

The radiological outcomes were as follows: of the 53 cases, solid union was observed in 50 cases (94.4%) and delayed union was seen in 3 cases (5.6%) at the 6th postoperative month, and complete union was identified in 52 cases (98.1%) at the last follow-up (Figs. 2 and 3). Radiolucency around the cage and pedicle screws was not observed in any of the cases at the last follow-up. Although instability caused by cage breakage was idenifited in 1 case (2%) at the 5th postoperative month, stability without further breakage was achieved at the 8th postoperative month. Trabecular continuity was not obvious at the last follow-up, but any radiolucency around the cage and screws and instability on the flexion-extension radiographs were not observed (Fig. 4). The intervertebral disc height significantly improved from 9.21mm preoperatively to 13.63 mm immediately postoperatively and it became 12.47 mm at the last follow-up. The mean increase of the intervertebral disc height of 3.26 mm from the preoperative measurement to the last follow-up examination was statistically significant (*p* = 0.009) (Fig. 5, Table 4).

The mean duration of surgery was 221.5 minutes (range, 140 to 320 minutes) for single-level fusion, 258.9 minutes (range, 200 to 440 minutes) for two-level fusion and 353.3 minutes (range, 290 to 410 minutes) for three-level fusion. The mean hemorrhage volume was 933.3 ml for single-level fusion, 964.2 ml for two-level fusion and 1,011.6 ml for three-level fusion. The mean hospitalization period was 14.5 days (range, 7 to 28 days).

Immediately after surgery, a case of cauda equina syndrome that occurred as a complication of hematoma formation was treated with removal of the hematoma. Although cage breakage was observed in 1 case, the radiography revealed no instability at the last follow-up.

## DISCUSSION

PLIF was designed to reduce the pain resulting from nerve compression and to secure the stability of the surgical constructs. The minor symptoms of such degenerative lumbar diseases as spinal stenosis and spondylolisthesis improve with conservative treatments in most cases. However, when the symptoms of these diseases such as back pain, radiating pain in the lower limb and neurogenic claudication severely restrict a person's daily activities, then surgical options should be taken into consideration.^1^,^8^-^11^,^13^,^18^,19)

Interbody fusion is one of the most common types of vertebral body fusions, and this is regarded as the most recommendable biomechanical technique. Particularly, the popularity of interbody fusion with using a cage has prompted the invention of various cages, which also led to the advancement of PLIF techniques.^13^,^20^,21) Posterolateral fusion involves the risk of muscle fibrosis caused by the extensive release of muscles adjacent to the transverse process, and the loss of blood and postoperative wound infection due to a lengthened operative time. In contrast, interbody fusion was advantageous for increasing the fusion rate and reducing the extensive muscle release around the transverse process with the fusion being performed at the level of the spinal compression, and obtained early stability and a high rate of fusion following PLIF with the use of pedicle screws for fixation.^22^-25) In this study, there were no complications such as infection that developed following PLIF with using pedicle screws and muscle release around the transverse process. In addition, early stability was obtained in many cases and satisfying clinical results and solid fusion union were achieved at the last follow-up.

There are three common types of PLIF techniques: one involves bilateral laminectomy and implantation of two cages, another involves unilateral laminectomy and implantation of two cages and the other involves unilateral laminectomy and implantation of one cage.^26^-28) The first and the last techniques have both been recently reported to be conducive to postoperative stability of the vertebral body. Oxland and Lund29) reported that single-cage PLIF provided high stability in flexion, that the supplementary use of pedicle screws improved the stabilization in all directions and that the two-cage PLIF might increase risk of damage to the bilateral nerve roots. Zhao et al.^20^,30) documented that single-cage PLIF was easier to perform than two-cage PLIF. Particularly, retraction of the nerve roots and the dura mater of the asymptomatic side could be avoided with unilateral placement of a cage in patients with unilateral sciatica, and the supplementry use of pedicle screws also allowed immediate postoperative stabilization. They also added that single-cage PLIF was advantageous in reducing the blood loss, the operative time and the hospital stay. In this study, single-cage PLIF minimized the damage to the posterior structures while providing proper decompression, high stability and a remarkable fusion rate, and the cost of an additional cage could be saved.

According to the biomechanical comparison of single-cage PLIF and two-cage PLIF by Chiang et al.,1) while both techniques result in a similar level of flexion of the spine, the former procedure additionally requires a bone graft. They postulated that a single cage that had a small implant-vertebral contact area led to an increase in the load of contact compression, which eventually raises the risk of cage subsidence, migration and breakage, and it increases the compression load on adjacent discs. In our study, the intervertebral contact surface could be enlarged by transplanting a bone graft anterior to the cage, which prevented the occurrence of the problems associated with single-cage PLIF. In addition, the graft anterior to the cage was an efficient indicator of boney union in spite of the radiopacity of the involved cages.

An autogenous bone graft can be accomplished with tricortical, bicortical and monocortical bone, cancellous bone, a morselized local bone graft obtained from decompression and transplantation of 3-5 blocks of morselized local bone obtained from decompression into the intervertebral discs. An iliac crest bone graft facilitates rapid bone union, but it increases the risk of donor site pain, excessive blood loss, donor site infection and pelvic fracture, and there is another skin incision and the operative time is lengthened. In contrast, a local bone graft shortens the operative time and blood loss because a bone harvesting procedure is not necessary and so there are no problems associated with any donor site, but a local bone graft is disadvantageous for the boney union due to its poor bone quality, as compared with the iliac crest bone graft. In this study, the lamina, the articular process and the spinous process obtained from decompression were morselized and then transplanted at the anterior portion of the intervertebral disc space and then a cage filled with local morselized bone was inserted. With these procedures, we could obtain excellent boney union and we reduced the operative time and blood loss.

In conclusion, this study used unilateral PLIF, a local morselized bone graft was placed anterior to the cage and a single cage filled with the graft was inserted. This technique produced satisfying clinical outcomes and radiological outcomes such as maintaining the proper intervertebral disc space, good boney union, rigid stability and a high fusion rate.

## Figures and Tables

**Table 1:**
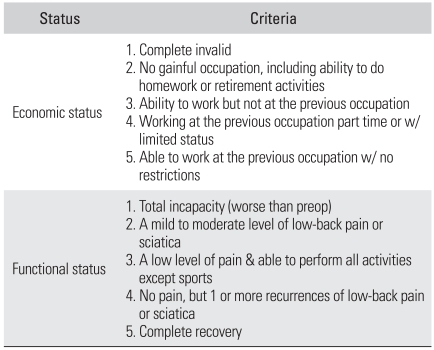
The Prolo Functional Economic Outcome Rating Scale

**Table 2:**
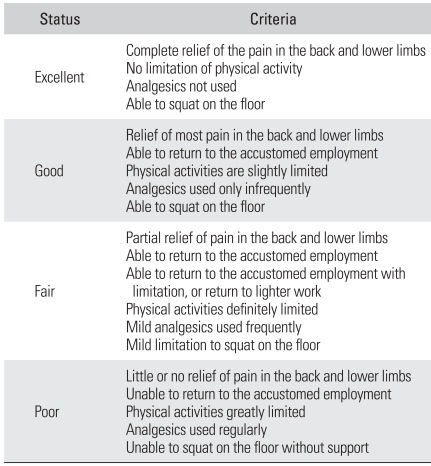
Criteria for the Clinical Results (by Kim & Kim)16)

**Table 3:**
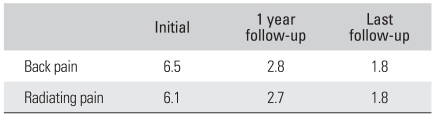
The Mean Pain Index (Visual Analogue Scale) during Following-up (*p*-value = 0.0001)

**Table 4:**
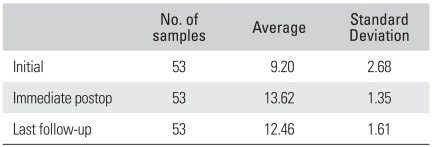
Changes of the Disc Height during Following-up (*p*-value = 0.009)
